# Seroprevalence of Hepatitis B Virus Infection and Its Associated Factors Among Blood Donors in Yemen

**DOI:** 10.21315/mjms2021.28.5.5

**Published:** 2021-10-26

**Authors:** Naif Tawfeq al-nwany, Norfazilah Ahmad, Azmawati Mohammed Nawi, Mohd Rohaizat Hassan, Rozita Hod, Mazni Baharom

**Affiliations:** 1 Department of Community Health, Faculty of Medicine, Universiti Kebangsaan Malaysia, Kuala Lumpur, Malaysia; 2 Faculty of Medicine and Health Sciences, University of Science and Technology, Sana’a, Yemen

**Keywords:** hepatitis B virus, prevalence, blood donors, Yemen

## Abstract

**Background:**

In limited-resource countries, the prevalence of hepatitis B virus (HBV) infection can be measured using data taken from blood donors. This study aimed to determine the HBV infection seroprevalence and its associated factors among blood donors in Sana’a Governorate, Yemen.

**Methods:**

A cross-sectional study was conducted on 500 people who donated blood at hospitals located in Sana’a between August and October 2016. The volunteers were aged 18–65 years old and were recruited using convenience sampling. A set of questionnaires was administered through face-to-face interviews. Blood samples from each donor were tested for hepatitis B surface antigens using enzyme-linked immunosorbent assay.

**Results:**

The overall seroprevalence of HBV infection in the blood donors was 2.6%. Participants with a history of dental treatment had 7.80 higher odds (95% confidence interval [CI]: 2.26, 26.71; *P* = 0.002) for HBV infection. Respectively, blood donors who had history of malaria infection or cupping therapy had 7.48 (95% CI: 1.75, 31.82; *P* = 0.010) and 7.32 (95% CI: 1.72, 30.83; *P* = 0.010) greater odds of HBV infection.

**Conclusion:**

The seroprevalence of HBV infection in Sana’a is lower than in other governorates in Yemen. Stakeholders could focus on a history of dental procedure, malaria infection and cupping treatment when strategising about HBV infection prevention and control among blood donors.

## Introduction

The World Health Organization (WHO) reported that hepatitis B virus (HBV) and hepatitis C virus (HCV) infections were responsible for 96% of all hepatitis mortality (1). An increasing trend of HBV deaths is predominated by primary hepatocellular carcinoma, followed by liver cirrhosis (1). The WHO also found that the rates of HBV infection in the general population globally and in the Eastern Mediterranean Region were 3.5% and 3.3%, respectively. Meanwhile, a meta-analysis of 66 studies from the WHO Eastern Mediterranean Region (EMRO) and Middle Eastern countries published between 1 January 2000 and 31 August 2015 discovered that the pooled prevalence of hepatitis B surface antigen (HBsAg) in blood donors was 2.03% (2). Moreover, the prevalence rates in the EMRO and Middle Eastern countries were 1.99% and 1.62%, respectively. More recent studies within the region indicated that the seroprevalence was lowest in Saudi Arabia (0.28%) (3) and highest in Jordan (4) and Iraq (5) (0.52% and 2.30%, respectively). Among the identified factors associated with HBV infection were increased age (6–7), being married, having a lower educational level, having specific occupations, family history of HBV infection and lack of immunisation (7).

Most Middle Eastern countries are developing nations with sub-optimal health care infrastructures and Yemen is one of the least developed; it has very limited resources (8). Yemen has been the site of political conflict since 2014; the violence has steadily escalated since, leading to one of the worst humanitarian crises today. The country’s instability has made obtaining the most reliable epidemiological data on HBV infection among the general population in Yemen very challenging. Data obtained from blood donors have been used as indicators of the HBV burden in countries similar to Yemen; that said, the data must be interpreted with caution, as the actual prevalence is likely underestimated (9).

Sana’a, the capital of Yemen, is an epicentre of this turmoil and remains trapped in a cycle of poverty and disease. Voluntary blood donors are the safest pool of blood donors, as they typically have better health-seeking behaviours than other blood donor groups (10). The region’s voluntary blood donors have played a crucial role in maintaining adequate blood supply for patients in need of transfusions (11). Getting the most updated information on HBV infection and its associated factors among blood donors in Sana’a is crucial, as the most recent measures were taken over a decade ago (12–13). The current state of the country does not enable screening of high-risk groups and large-scale screening has proved to be an impossible task. Thus, the present study aimed to determine the prevalence of HBV infection and the associated factors among voluntary blood donors in Sana’a.

## Methods

### Study Population

A cross-sectional study was conducted among volunteer blood donors who attended the blood banks in hospitals in Southern Sana’a Governorate, Yemen between August 2016 and October 2016. A total of 500 blood donors agreed to participate in the study and were recruited through convenience sampling. The sample size was calculated using Power and Sample Size Calculation (PS) software (14) with a two-sided 5% significance level, 80% power and reference to a previous local study by Al-Waleedi and Khader (15). These authors reported an HBV infection prevalence of 11.8% and 3.7% among blood donors with and without history of cupping therapy, respectively. The present study recruited an additional 20% above the calculated minimum of 408-person sample size to compensate for non-response.

Healthy volunteer blood donors aged 18 years old–65 years old were included in this study. The exclusion criteria were blood donors with haemoglobin level < 12 mg/dL, weight < 50 kg, recent history of surgery, history of blood donation < 3 months and history of cigarette smoking < 12 h.

Ethical approval was obtained from the Research and Ethics Committee, Faculty of Medicine and Health Sciences, University of Science and Technology, Sana’a; the Yemen Ministry of Health; and the hospitals’ directors. Written consent was obtained from all the participants, and they were assured the study would maintain data confidentiality. All data generated and analysed during this study that underlie the results have been included in this article.

### Data Collection

Data were collected using a structured self-administered questionnaire; the first author conducted face-to-face interviews with all participants who could not read and write. The questionnaire consisted of three sections: i) Section A measured sociodemographic characteristics (age, sex, marital status, residence, level of education, type of occupation and income); ii) Section B measured clinical factors (family history of jaundice, history of surgical procedure, blood transfusion, dental treatment, hepatitis B vaccination and history of physician-diagnosed malaria infection) and iii) Section C measured behavioural characteristics (shaving using razors/sharp gadgets, history of cupping therapy and chewing khat). Seroprevalence was measured from blood samples collected from each donor. The blood samples were tested for HBsAg in the respective hospital virology laboratory using an enzyme-linked immunosorbent assay (ELISA) (Progen, Germany) according to the manufacturer’s instructions. HBV infection was defined as blood samples that were positive for HBsAG.

### Statistical Analysis

Statistical analyses were performed using SPSS version 21.0 software (IBM Corp., Armonk, NY, USA). Categorical data are described as frequency (*n*) and percentage (%). Type of occupation was recategorised to ease univariable analysis. Simple logistic regression analysis was conducted to select variables for further analysis; the crude odd ratios (cOR) and the respective 95% confidence intervals (CI) were also obtained. Adjusted analysis was conducted using backward stepwise multiple logistic regression analysis to determine the final factors associated with HBV infection; the adjusted ORs (aORs) and their respective 95% CI were obtained. Significance level was set at *P* < 0.05.

## Results

A total of 500 volunteer blood donors participated in this study. Table 1 depicts the participants’ characteristics. Half of the participants (50.2%) were in the 18 years old–30 years old age group, the majority were male (94.8%), married (66%) and resided in Sana’a (63.8%). A total of 40.2% and 45.2% of the participants had attained primary and secondary education level, respectively. Regarding the type of occupation, manual workers predominated (20%), followed by military personnel (17.2%) and farmers (16.2%).

The seroprevalence of HBV infection among the blood donors was 2.6% (13/500). Of the 16 studied factors, bivariable analysis showed that three factors were significantly associated with HBV infection — namely, blood donors with a history of dental treatment, malaria infection and cupping therapy (Table 2). Table 3 illustrates that these three factors remained significantly associated with HBV infection after multivariable analysis. Participants with a history of dental treatment had 7.80 higher odds for HBV infection (95% CI: 2.26, 26.71; *P* = 0.002). Further, volunteer blood donors who had a history of malaria infection and cupping therapy had 7.48 (CI: 1.75, 31.82; *P* = 0.010) and 7.32 (95% CI: 1.72, 30.83; *P* = 0.010) greater odds of HBV infection, respectively.

## Discussion

HBV infection is a common public health problem — especially in developing countries — and it is associated with serious consequences such as liver cirrhosis and hepatocellular carcinoma (16). The prevalence of HBV infection in the present study was similar to that of a study conducted at Al-Thawra General Hospital in Sana’a (2.1%) (17). Despite the similar prevalence, the former study (17) did not explicitly describe whether their study population contained both sexes.

That said, prevalence rate of the present study was lower than previous studies conducted more than a decade ago among blood donors in Sana’a by Al-Nassiri and Raja’a (13) and Sallam et al. (12), which were 7.4% and 15.1%, respectively. The difference in the prevalence rate reported by Al-Nassiri and Raja’a (13) could be due to the wide age range involved in the study (1 month old–95 years old). Meanwhile, the difference in the prevalence rate reported by Sallam et al. (12) was probably because their study population involved only men, whereas the present study involved both sexes. Moreover, the reduced prevalence rate of HBV infection over the decade could be due to the availability of hepatitis B vaccination programmes (18). This warrants further evaluation, as the country has since plunged into conflict and resources became limited; the reduced prevalence is rising due to inadequate vaccine coverage, lack of prevention and screening strategies and insufficient or no access to treatment (19). The present study’s prevalence rate was also lower compared to different regions in the country, such as those found in Aden (5.1%) (15) and Hajjah (9.8%) (20). These dissimilar prevalence rates could be explained by the different study populations, geographical locations and availability of vaccination programmes.

The present results indicate that a history of dental treatment and cupping are significantly associated with HBV infection. This is in line with a previous local study in Aden (15), which indicated that blood donors with a history of dental treatment and cupping were at a higher risk for HBV infection. Despite the former and present study being conducted almost a decade apart, these two factors remain significantly associated with HBV infection. This may indicate that both dental clinics and cupping centres do not have proper infection control procedures. Previous studies from neighbouring countries have also demonstrated similar associations. Studies conducted in Iraq (21) and the Kingdom of Saudi Arabia (22) have shown that a history of dental treatment was associated with almost two times greater odds for HBV infection (aOR: 2.3; *P* = 0.030) and (aOR: 1.98; 95% CI: 1.15, 3.41; *P* = 0.013), respectively.

A study of the general population in Iran reported that a history of traditional cupping (Hijamat) was significantly associated with HBV infection (*P* = 0.005); however, this lost its significance after an adjusted analysis (23). Traditional cupping had been associated with bloodborne infections, usually due to unsterile treatment procedures (24). Indeed, some country namely, Saudi Arabia rejects potential blood donors if they have a history of cupping (25). Other studies have shown the therapeutic benefit of cupping for reducing hepatitis B viral load (if it is practiced under sterile conditions) (26).

Similar to another study conducted in Yemen (15), the present results show that a history of malaria is significantly associated with HBV infection. Many previous studies have reported malaria and HBV co-infections among various subpopulations, including immigrants (27), pregnant women (28) and blood donors (29). This co-infection could be associated with the natural progression of both diseases, as it has been suggested that these two infections share some of their developmental stages within the liver, causing significant changes in liver function (30). Nevertheless, the significant association in the present study needs to be interpreted and compared with caution, as the findings are based on a self-reported history of malaria infection, whereas the previous studies reported malaria infection based on diagnostic tests.

This study has several limitations. First, the study design limits cause-and-effect determination, as the sampling restricts study generalisation and there is potential information bias because most of the potential factors are based only on self-reporting. Second, the estimate of effects from this study needs to be interpreted with caution due to the wide confidence intervals. This was possibly caused by the sample size, which was calculated using just one of the factors and was not large enough to examine associations between other factors and HBV infection prevalence. Third, HBV seroprevalence in this study was detected using HBsAg, which can only determine whether the blood donor had the infection at the time of donation. Tests that measure HBsAg antibodies (anti-HBs), hepatitis B core antigens (anti-HBc), hepatitis B e antigens (HBeAg) and anti-Hbe to distinguish between infection and immunity could not be conducted due to limited resources.

Notably, chewing khat was included as one of the studied behavioural factors. Although there was no direct association between chewing khat and HBV infection, the findings of this study could be used to generate hypotheses for further studies, such as mediation analysis. Previous literature indicated an association between chewing khat, HIV-risk behaviours (31, 32) and the occurrence of self-reported sexually transmitted infection (32). This warrants further investigation on the direct and indirect association of chewing khat and HBV infection. Furthermore, this study was conducted in the early phase of Yemen’s conflict; its findings can be a baseline for further studies, especially those based on the Global Health Sector Strategy (33) attempting to eliminate viral hepatitis.

## Conclusion

The prevalence of HBV infection in Sana’a is relatively lower than that of other governorates in Yemen. History of dental procedure, malaria infection and cupping treatment could be useful when creating strategies for HBV infection prevention and control among blood donors.

## Figures and Tables

**Table 1:** Characteristics of the blood donors

Factor	HBV infection	

	Positive *n* (%) *n* = 13	Negative *n* (%) *n* = 487
Sociodemographic		
Age (years old)		
18–30	6 (2.4)	245 (97.6)
31–65	7 (2.8)	242 (97.2)
Sex		
Male	13 (2.7)	461 (97.3)
Female	0	26 (100)
Marital status		
Married	10 (3.0)	320 (97.0)
Single	3 (1.8)	167 (98.2)
Residence		
Sana’a	8 (4.4)	173 (95.6)
Other	5 (1.6)	314 (98.4)
Level of education		
Illiterate	2 (5)	38 (95)
Primary	3 (1.5)	198 (98.5)
Secondary	7 (3.1)	219 (96.9)
Tertiary	1 (3)	32 (97)
Type of occupation		
Manual worker	5 (5)	95 (95)
Military	2 (2.3)	84 (97.7)
Driver	2 (4.2)	46 (95.8)
Student	0	46 (100)
Farmer	0	81 (100)
Housewife	0	9 (100)
Nurse	1 (11.1)	8 (88.9)
Other	3 (2.5)	118 (97.5)
Income (USD) (*n* = 492)		
≤ 70	3 (1.7)	174 (98.3)
> 70	10 (3.2)	305 (96.8)
Clinical factors		
Family history of jaundice		
No	12 (2.5)	471 (97.5)
Yes	1 (5.9)	16 (94.1)
Surgical history		
No	12 (2.6)	441 (97.4)
Yes	1 (1.2)	46 (97.9)
Blood transfusion history		
No	12 (2.5)	474 (97.5)
Yes	1 (7.1)	13 (92.9)
Dental treatment history		
No	7 (1.6)	441 (98.4)
Yes	6 (11.5)	46 (88.5)
Hepatitis B vaccination		
No	13 (2.9)	436 (97.1)
Yes	0	51 (100)
Malaria infection history		
No	9 (1.9)	460 (98.1)
Yes	4 (12.9)	27 (87.1)
Behavioural		
Shaving using razor/sharp gadget		
No	10 (2.3)	428 (97.6)
Yes	3 (4.8)	59 (95.2)
Cupping therapy		
No	9 (1.9)	459 (98.1)
Yes	4 (12.5)	28 (87.5)
Chewing khat		
No	1 (1.3)	76 (98.7)
Yes	12 (2.8)	411 (97.2)

Note: USD = US Dollar

**Table 2:** Preliminary model of factor associated with HBV infection among the blood donors

Factor	cOR (95% CI)*	*P-*value
Sociodemographic		
Age (years old)		
18–30	1	
31–65	1.18 (0.39, 3.57)	0.768
Marital status		
Single	1	
Married	1.74 (0.47, 6.41)	0.400
Residence		
Other	1	
Sana’a	2.90 (0.94, 9.01)	0.054
Level of education		
Tertiary	1	
Secondary	1.02 (0.12, 8.59)	0.983
Primary	0.48 (0.05, 4.81)	0.528
Illiterate	1.68 (0.15, 19.44)	0.673
Type of occupation		
Other	1	
Manual worker	2.07 (0.48, 8.88)	0.318
Non-manual worker	0.79 (0.16, 3.97)	0.943
Military	0.54 (0.09, 3.28)	0.560
Income (USD) (*n* = 492)		
> 70	1	
≤ 70	0.53 (0.14, 1.94)	0.326
Clinical factors		
Family history of jaundice		
No	1	
Yes	2.45 (0.30, 20.03)	0.387
Surgical history		
No	1	
Yes	0.80 (0.10, 6.28)	0.831
Blood transfusion history		
No	1	
Yes	3.04 (0.37, 25.14)	0.280
Dental treatment history		
No	1	
Yes	8.22 (2.65, 25.49)	< 0.001
Malaria infection history		
No	1	
Yes	7.57 (2.19, 26.17)	< 0.001
Behavioural		
Shaving using razor/sharp gadget		
No	1	
Yes	2.18 (0.58, 8.14)	0.237
Cupping therapy		
No	1	
Yes	7.29 (2.11, 25.13)	< 0.001
Chewing khat		
No	1	
Yes	2.12 (0.28, 17.31)	0.435

Notes:

* simple logistic regression; 1: reference group; USD: US Dollar

**Table 3:** Final model of factor associated with HBV infection among the blood donors

Factor	aOR (95% CI)**	*P-*value
Dental treatment history		
No	1	
Yes	7.80 (2.26, 26.71)	0.002
Malaria infection history		
No	1	
Yes	7.48 (1.75, 31.82)	0.010
Cupping therapy		
No	1	
Yes	7.32 (1.72, 30.83)	0.010

Notes:

** multiple logistic regression (Backward LR); 1: reference group
